# Plasma amino acids in major depressive disorder: between pathology to pharmacology

**DOI:** 10.17179/excli2023-6767

**Published:** 2024-01-04

**Authors:** Omar Gammoh, Alaa A. A. Aljabali, Murtaza M. Tambuwala

**Affiliations:** 1 Department of Clinical Pharmacy and Pharmacy Practice; Faculty of Pharmacy, Yarmouk University Irbid 21163, PO BOX 566, Jordan; 2 Department of Pharmaceutics and Pharmaceutical Technology; Faculty of Pharmacy, Yarmouk University Irbid 21163, PO BOX 566, Jordan; 3 Lincoln Medical School, University of Lincoln, Brayford Pool Campus, Lincoln LN6 7TS, United Kingdom

**Keywords:** major depressive disorder (MDD), plasma amino acids, pathophysiology, biomarkers, tryptophan metabolism

## Abstract

Addressing the formidable challenge posed by the development of effective and personalized interventions for major depressive disorder (MDD) necessitates a comprehensive comprehension of the intricate role that plasma amino acids play and their implications in MDD pathology and pharmacology. Amino acids, owing to their indispensable functions in neurotransmission, metabolism, and immune regulation, emerge as pivotal entities in this intricate disorder. Our primary objective entails unraveling the underlying mechanisms and unveiling tailored treatments through a meticulous investigation into the interplay between plasma amino acids, MDD, and pharmacological strategies. By conducting a thorough and exhaustive review of the existing literature, we have identified pertinent studies on plasma amino acids in MDD, thereby uncovering noteworthy disturbances in the profiles of amino acids among individuals afflicted by MDD when compared to their healthy counterparts. Specifically, disruptions in the metabolism of tryptophan, phenylalanine, and tyrosine, which serve as precursors to essential neurotransmitters, have emerged as prospective biomarkers and critical contributors to the pathophysiology of depression. Amnio acids play an essential role in MDD and could represent an attractive pharmacological target, more studies are further required to fully reveal their underlying mechanisms.

## Major Depressive Disorder (MDD)

Addressing the formidable challenge of developing effective and personalized interventions for major depressive disorder (MDD) necessitates a comprehensive understanding of the intricate role of plasma amino acids and their implications in MDD pathology and pharmacology (Ogawa et al., 2018[50]). Amino acids, due to their indispensable functions in neurotransmission, metabolism, and immune regulation, emerge as pivotal entities in this complex disorder. Our primary aim is to unravel the underlying mechanisms and reveal customized treatments by investigating the interplay between plasma amino acids, MDD, and pharmacological strategies (Zaki et al., 2017[86]). To accomplish this objective, we conducted an exhaustive review of the literature, utilizing various databases and employing precise search terms to identify relevant studies on plasma amino acids in MDD. Our analysis uncovered noteworthy disturbances in the profiles of plasma amino acids in individuals affected by MDD compared to their healthy counterparts. Specifically, disruptions in tryptophan, phenylalanine, and tyrosine metabolism, which are precursors to vital neurotransmitters, have emerged as prospective biomarkers and critical contributors to the pathophysiology of depression.

While duly acknowledging the inherent challenges, we also address the limitations associated with relying solely on amino acid biomarkers. We recognize the multifaceted nature of depression, encompassing genetic factors, environmental influences, and intricate neurochemical dynamics that require meticulous scrutiny. Factors such as inter-individual variability, dietary influences, and comorbid conditions further complicate the interpretation of amino acid biomarkers. Our exploration transcends conventional therapeutics, paving the way for precision medicine in the realm of depression. Harnessing non-invasive techniques for profiling amino acids facilitates the identification of biomarkers for precise diagnosis and tailored treatments. Moreover, insights into the dynamics of amino acids may unveil innovative drug targets, potentially revolutionizing intervention strategies. In conclusion, our comprehensive analysis of plasma amino acids in MDD significantly advances our comprehension of this intricate disorder. By bridging the gap between the pathophysiology of MDD and its pharmacology, we unlock the potential of amino acid biomarkers, uncover new mechanisms, and ultimately transform the treatment landscape for individuals suffering from MDD. Through meticulous searches across various databases, incorporating specific search terms, we present a robust overview of the current literature on plasma amino acids in MDD, ensuring the inclusion of pertinent studies.

MDD presents substantial hurdles in the development of effective and individualized interventions. Exploring the role of plasma amino acids and their implications for MDD pathology and pharmacology offers a promising avenue for advancing our understanding and treatment of this complex disorder (Peplinska-Miaskowska et al., 2022[55]). Amino acids, essential building blocks of proteins, play critical roles in neurotransmission, metabolism, and immune regulation (Dalangin et al., 2020[14]). Our objective is to unravel the underlying mechanisms and reveal personalized treatments by examining the interplay between plasma amino acids, MDD, and pharmacological strategies. To comprehensively identify relevant studies on plasma amino acids in MDD, we conducted an extensive literature review utilizing various databases and specific search terms. The analysis of these studies uncovers altered plasma amino acid profiles in individuals with MDD compared to healthy controls. Of particular interest are disruptions in tryptophan, phenylalanine, and tyrosine metabolism, as these amino acids serve as precursors for neurotransmitters associated with mood regulation. These findings suggest that dysregulation in amino acid metabolism and neurotransmitter synthesis plays a critical role in the pathophysiology of depression and may serve as valuable biomarkers (Lanser et al., 2020[33]; Richard et al., 2009[61]). 

While the potential of amino acids as biomarkers is intriguing, it is important to acknowledge the limitations and challenges associated with relying solely on these markers (Skelley et al., 2005[71]) as shown in Table 1(Tab. 1). Depression is a multifactorial condition influenced by genetic, environmental, and neurochemical factors, necessitating a comprehensive examination. Factors such as inter-individual variability, dietary influences, and comorbid conditions can confound the inter-pretation of amino acid levels in MDD (Cacabelos, 2007[10]). Furthermore, the dynamic nature of amino acid metabolism requires longitudinal studies to capture fluctuations over time and their relationship to treatment response and disease progression. In contrast to traditional therapeutics like selective serotonin reuptake inhibitors (SSRIs) and serotonin-norepinephrine reuptake inhibitors (SNRIs) that primarily target neurotransmitter systems, investigating the potential of plasma amino acids offers distinct advantages (Ansone et al., 2021[2]). Amino acid profiling provides a non-invasive and easily accessible approach to identifying biomarkers, enabling more precise diagnosis and individualized treatment selection. By measuring amino acid levels in plasma, clinicians may identify subtypes of depression with distinct underlying mechanisms and tailor treatment approaches accordingly. Moreover, an in-depth understanding of amino acid dynamics may reveal novel drug targets, revolutionizing interventions. By targeting specific enzymes or transporters involved in amino acid metabolism, it may be possible to modulate neurotransmitter synthesis and restore the neurochemical balance in MDD (Woo et al., 2015[79]).

The novelty of research on plasma amino acids in MDD lies in its potential to bridge the gap between pathology and pharmacology. Unraveling the underlying mechanisms linking dysregulated amino acid metabolism to depressive symptomatology holds promise for identifying novel therapeutic strategies. This approach moves beyond the traditional paradigm of targeting specific neurotransmitter systems, emphasizing the need for a more comprehensive understanding of depression. Additionally, longitudinal monitoring of amino acid profiles may allow clinicians to track treatment responses and guide individualized treatment adjustments, ultimately enhancing patient outcomes. In conclusion, investigating plasma amino acids in the context of MDD represents a promising avenue for advancing our understanding of the disorder's pathophysiology and improving treatment outcomes. While acknowledging the complexities and challenges associated with this line of research, exploring amino acid biomarkers and their interplay with pharmacological interventions holds significant potential for precision medicine in depression. Further investigations are warranted to validate and expand upon the current knowledge, ultimately translating these findings into tangible benefits for patients suffering from MDD (Ong et al., 2021[51]).

## Rationale of Amino Acid Study in MDD

When examining the occurrence of plasma amino acid abnormalities in major depressive disorder (MDD), a substantial body of evidence indicates the presence of dysregulation in specific amino acids among individuals with MDD (Ogawa et al., 2018[50]). Tryptophan, an essential amino acid and precursor to serotonin, consistently exhibits significant reductions in individuals diagnosed with MDD compared to healthy controls. This decline in tryptophan levels likely contributes to serotonin deficiency, a characteristic feature of MDD and its associated symptoms (Liu et al., 2022[37]). Similarly, individuals with MDD display lower plasma levels of phenylalanine, another essential amino acid involved in neurotransmitter synthesis. Altered phenylalanine levels may influence the availability of neurotransmitters such as dopamine and norepinephrine, which have vital roles in regulating mood (Ho et al., 2023[22]).

Consistent findings from multiple studies also indicate abnormalities in plasma glutamate levels among individuals with MDD. Increased levels of plasma glutamate are observed in MDD patients, suggesting disrupted glutamatergic neurotransmission and potential implications for neuroinflammation, oxidative stress, and synaptic dysfunction associated with MDD (Inoshita et al., 2018[25]). Conversely, reduced plasma glutamine levels have also been noted in MDD patients, further indicating disturbances in glutamatergic metabolism. Additionally, branched-chain amino acids (BCAAs), including leucine, isoleucine, and valine, have been implicated in plasma amino acid alterations in MDD. Elevated plasma levels of BCAAs are observed in individuals with MDD, potentially contributing to insulin resistance, disrupted amino acid metabolism, and effects on neurotransmission that may influence mood regulation and the severity of depressive symptoms (Inoshita et al., 2018[25]).

It is vital to acknowledge that the prevalence of plasma amino acid alterations in MDD may vary due to factors such as the heterogeneity of the disorder, patient characteristics, and methodological variations across studies. Nevertheless, the consistent findings of altered amino acid levels across various investigations underscore the potential utility of amino acid profiling as a biomarker and the involvement of amino acid dysregulation in the pathophysiology of MDD. Further research is necessary to establish causality, elucidate underlying mechanisms, and validate the clinical utility of plasma amino acid profiling in MDD. Large-scale longitudinal studies involving diverse populations are warranted to confirm the prevalence of plasma amino acid alterations and their potential as diagnostic or predictive markers in the management of MDD (Ogawa et al., 2018[50]).

The prevalence of plasma amino acid alterations in MDD is substantiated by consistent findings of dysregulation in specific amino acids, such as tryptophan, phenylalanine, glutamate, glutamine, and BCAAs. These examples provide a clearer understanding of the prevalence and nature of plasma amino acid alterations in MDD. Future research should focus on expanding our knowledge of these alterations, unraveling their underlying mechanisms, and exploring their potential as diagnostic or therapeutic targets in MDD (Islam et al., 2020[26]).

## Impact of Amino Acids in MDD

Plasma amino acids play a significant role in the complex interplay between metabolic dysregulation and major depressive disorder (MDD), influencing various aspects of the disorder, including symptomatology, treatment response, and potential therapeutic interventions (Ogawa et al., 2018[50]). Understanding the impact of plasma amino acids on MDD is crucial for advancing our knowledge in this field. 

### Influence on depressive symptomatology

Plasma amino acids have notable influences on depressive symptomatology in individuals with MDD. Consistent evidence suggests that reduced plasma levels of tryptophan, an essential amino acid, and serotonin precursor, are associated with MDD. Tryptophan deficiency can lead to serotonin deficiency, a neurochemical abnormality commonly observed in MDD. These disruptions in tryptophan metabolism contribute to imbalances in mood regulation, resulting in persistent low mood, loss of interest, and feelings of guilt (Young and Leyton, 2002[84]).

Likewise, alterations in plasma levels of other essential amino acids, such as phenylalanine, can impact neurotransmitter availability and contribute to the severity of depressive symptoms. Phenylalanine plays a crucial role in the synthesis of neurotransmitters like dopamine and norepinephrine, which are vital for mood regulation. Dysregulation in phenylalanine levels may disrupt neurotransmitter balance, exacerbating depressive symptomatology. 

### Implications for treatment response

Plasma amino acid profiles have implications for treatment response in individuals with MDD, highlighting their potential as predictive markers and treatment targets. Low baseline levels of tryptophan have been associated with poor response to conventional antidepressant medications. This suggests that individuals with MDD characterized by tryptophan deficiency may require alternative treatment strategies, such as serotonin-targeting agents or adjunctive therapies aimed at restoring amino acid balance.

Exploring amino acid profiling as a tool for predicting treatment response holds promise. By examining baseline plasma amino acid levels, it may be possible to identify individuals who are more likely to respond favorably to specific treatment modalities. This personalized approach can improve treatment outcomes, optimize therapeutic interventions, and reduce the need for trial-and-error approaches. 

### Potential therapeutic interventions

The impact of plasma amino acids in MDD extends beyond their role as biomarkers or predictors of treatment response. It presents opportunities for developing innovative therapeutic interventions targeting metabolic pathways and amino acid dysregulation (Levine et al., 2000[34]).

Strategies focused on amino acid supplementation or modulation have shown promise in preclinical and clinical studies. By restoring or rebalancing specific amino acids, it may be possible to rectify the metabolic perturbations associated with MDD. Targeted interventions could involve the administration of amino acid precursors, or the modulation of enzymes involved in amino acid metabolism to restore neurotransmitter synthesis and promote mood stabilization. Furthermore, considering the bidirectional communication between the gut and brain, interventions aimed at modulating the gut microbiota hold potential. Preclinical studies have highlighted the connection between the gut microbiome, amino acid metabolism, and MDD (Parker and Brotchie, 2011[53]). Restoring a healthy gut microbiota through interventions like probiotics may positively impact plasma amino acid profiles, contributing to improved depressive symptoms. Plasma amino acids have a profound impact on various aspects of major depressive disorders. They influence depressive symptomatology, treatment response, and potential therapeutic interventions. By understanding the role of plasma amino acids, we can develop targeted strategies that address metabolic dysregulation and optimize treatment outcomes in individuals with MDD. Further research is necessary to unravel the underlying mechanisms and validate the clinical utility of plasma amino acid profiling in personalized management approaches for MDD (Rondanelli et al., 2011[62]).

## Challenges of Amino Acid Study in MDD

### Introduction

Despite the potential of plasma amino acids as markers and targets for major depressive disorder (MDD), several challenges must be addressed to comprehensively understand their role in the disorder's pathophysiology and management. This section aims to elucidate the challenges associated with plasma amino acids in MDD, including limitations in research methodologies, variability in findings, and the need for further investigation. 

### Methodological challenges

The study of plasma amino acids in MDD presents methodological challenges that can affect the accuracy and reliability of findings. Collection and analysis of plasma samples pose a significant challenge, as factors such as sample handling, storage conditions, and analytical techniques can introduce variability in amino acid measurements. Standardizing sample collection and analysis protocols is essential to minimize these challenges and ensure robust and comparable results across studies (Xu et al., 2012[82]).

Additionally, the dynamic nature of amino acid metabolism presents another challenge. Plasma amino acid levels can fluctuate due to factors like dietary intake, circadian rhythm, stress, and comorbid conditions. Capturing these fluctuations accurately and accounting for confounding factors pose challenges in studying the specific alterations associated with MDD. Longitudinal studies with frequent and standardized measurements are necessary to overcome these challenges and obtain a comprehensive understanding of the dynamic changes in plasma amino acid profiles in MDD. 

### Heterogeneity and variability

Major depressive disorder is a highly heterogeneous condition characterized by diverse symptomatology, etiological factors, and treatment response. This heterogeneity poses challenges in interpreting findings related to plasma amino acids (Trzaskowski et al., 2019[75]). Variability in amino acid levels among individuals with MDD can be influenced by factors such as age, sex, symptom severity, medication use, and comorbidities. Genetic, environmental, and physiological factors further contribute to this variability. To address these challenges, future research should consider factors contributing to heterogeneity and develop strategies to account for them. Large-scale studies that include diverse populations, consider potential confounders, and employ sophisticated statistical analyses can help elucidate the specific amino acid alterations relevant to MDD subtypes and guide personalized interventions (Price et al., 2009[57]). 

### Causality and mechanistic understanding

Establishing causality and unraveling the underlying mechanisms between plasma amino acids and MDD is a significant challenge. Although associations between altered amino acid profiles and MDD have been observed, the direction of causality remains unclear. Determining whether the observed amino acid alterations are a cause or consequence of the disorder is challenging. To address this challenge, prospective studies and experimental models are needed to explore the temporal relationship between plasma amino acids and the development of MDD. Integrating multi-omics approaches, such as metabolomics, genomics, and proteomics, can provide a more comprehensive understanding of the complex molecular interactions and pathways involved in MDD, shedding light on the mechanistic underpinnings of amino acid dysregulation (Baghai et al., 2018[3]).

### Clinical translation and treatment implications

Another challenge lies in the clinical translation of plasma amino acids into practical diagnostic tools and therapeutic interventions for MDD. While identifying potential biomarkers and treatment response predictors is promising, their utility in routine clinical practice is yet to be established. Developing robust and standardized assays for measuring plasma amino acids, along with the integration of machine learning algorithms, can enhance the diagnostic accuracy and predictive value of amino acid profiles. This can help identify MDD subgroups, predict treatment outcomes, and guide personalized treatment strategies (Batch et al., 2014[5]). 

### Conclusion

Addressing the challenges associated with plasma amino acids in major depressive disorder requires a multidisciplinary and collaborative approach to advance our understanding of their role in the disorder. Overcoming methodological limitations, addressing heterogeneity, elucidating causality, and translating findings into clinical practice are key areas for future research. By addressing these challenges, we can unlock the full potential of plasma amino acids as biomarkers and therapeutic targets, paving the way for personalized and effective management strategies for individuals with MDD (Simińska and Koba, 2016[70]).

## Future Aspects

While the existing literature has shed light on the role of plasma amino acids in major depressive disorder (MDD), there are several aspects that require further exploration to advance our understanding of the complex interplay between amino acid metabolism and MDD pathophysiology. Critically examining these aspects will help identify novel targets for therapeutic interventions and refine personalized treatment approaches. Here, we discuss key areas that necessitate additional research:

### Genetic variations and amino acid dysregulation

Although genetic factors play a crucial role in MDD susceptibility, the specific genetic variations influencing amino acid metabolism in the context of MDD remain understudied. Future research should focus on identifying specific genes or genetic variants associated with amino acid dysregulation in MDD. Understanding the functional implications of these genetic variations will provide insights into the underlying molecular mechanisms and potential therapeutic targets. 

### Exploring the gut-brain axis

Mounting evidence suggests that the gut microbiota and their metabolites play a significant role in influencing amino acid metabolism and mental health. However, the precise mechanisms through which the gut-brain axis impacts amino acid dysregulation in MDD require further investigation. Future studies should aim to elucidate how gut microbiota composition and their metabolites influence amino acid metabolism, neuroinflammation, and neurotransmitter synthesis in MDD, offering potential avenues for therapeutic intervention (Luna and Foster, 2015[38]; Ogawa et al., 2018[50]) as summarized in Table 2(Tab. 2) (References in Table 2: Baranyi et al., 2016[4]; Behera et al., 2022[7]; Kasakin et al., 2020[29]; Liu et al., 2022[36]; Sandstrom et al., 2008[64]; Veldic et al., 2019[76]; Wu et al., 2016[81]).

### Biomarker discovery

While the potential of plasma amino acids as biomarkers for MDD has been proposed, more research is needed to validate their diagnostic accuracy and clinical utility. Future studies should employ rigorous methodologies to identify specific amino acid profiles that reliably differentiate individuals with MDD from healthy controls. Additionally, investigating the specificity and sensitivity of these biomarkers in differentiating MDD from other psychiatric disorders would enhance their clinical applicability. 

### Augmentation strategies

Amino acids have shown promise as adjunctive therapies to conventional antidepressants. However, the specific amino acids, dosages, and treatment protocols warrant further investigation. Future research should conduct well-designed clinical trials to evaluate the efficacy, optimal dosing, and duration of amino acid augmentation in MDD treatment. Additionally, examining the underlying mechanisms of action and potential synergistic effects with existing pharmacotherapies will contribute to the development of personalized treatment strategies (Ding et al., 2014[18]).

### Gender differences

MDD exhibits notable gender differences in prevalence, symptomatology, and treatment response. Exploring gender-specific variations in plasma amino acid profiles and their impact on MDD pathophysiology is crucial for tailoring treatment approaches. Future studies should investigate gender-specific differences in amino acid metabolism, considering hormonal influences and their potential implications for treatment response and personalized medicine. 

### Longitudinal studies

Longitudinal studies are vital for understanding the dynamic nature of amino acid dysregulation in MDD. Investigating changes in plasma amino acid profiles over time and their correlation with symptom severity, treatment response, and relapse risk will provide insights into the disease course and the effectiveness of therapeutic interventions. Future research should prioritize longitudinal study designs to capture temporal changes and establish causal relationships (Schuch et al., 2014[69]).

In conclusion, while significant progress has been made in unraveling the role of plasma amino acids in MDD, several aspects require further exploration. By conducting in-depth investigations into genetic variations, the gut-brain axis, biomarker discovery, augmentation strategies, gender differences, and longitudinal studies, we can expand our knowledge of amino acid metabolism in MDD and develop more targeted and effective interventions. Continued collaborative efforts among researchers, clinicians, and industry partners will propel the field forward, ultimately improving outcomes for individuals affected by MDD (Xu et al., 2012[82]).

## Plasma Amino Acids

### Tryptophan

The role of tryptophan (Trp) in major depressive disorder (MDD) is of significant interest. The majority of circulating Trp is bound, with only a small fraction being free and capable of crossing the blood-brain barrier. Once in the brain, Trp serves as a precursor for serotonin, a neurotransmitter involved in mood regulation. Animal models have demonstrated that Trp depletion in the diet leads to reduced brain serotonin levels and dysfunction (Bell et al., 2001[8]). Studies involving humans have shown that Trp depletion diets can decrease cerebrospinal fluid (CSF) Trp levels by up to 90% (Carpenter et al., 1998[11]). Numerous studies in individuals with MDD have consistently reported decreased plasma levels of Trp (Krishnan and Nestler, 2008[30]; Ogawa et al., 2014[49]). For example, in recently diagnosed and medication-naïve depressed patients, Trp plasma levels were significantly lower compared to controls (Zheng et al., 2012[87]). A meta-analysis examining the role of 12 amino acids in depression concluded that Trp deficiency was strongly associated with severe depression (Huang et al., 2021[24]). This may be explained by the low-grade inflammation cascade mediated by cytokines, which activates the tryptophan-kynurenine pathway implicated in MDD pathology (Dantzer et al., 2011[15]; Schiepers et al., 2005[67]).

Treatment interventions can also impact plasma Trp levels. For instance, studies have shown that 24 hours after electroconvulsive therapy (ECT), plasma Trp levels were increased only in individuals who responded to the treatment (Hoekstra et al., 2001[23]). Another study confirmed this finding, demonstrating that plasma levels of total Trp continued to be increased 2-24 hours after ECT administration (Palmio et al., 2005[52]). Antidepressant medications, such as selective serotonin reuptake inhibitors (SSRIs), act on serotonin receptors to increase extracellular serotonin levels, reducing depressive symptoms. Treatment with fluvoxamine, a commonly used SSRI, has been shown to increase plasma Trp levels after 4 weeks of therapy (Mauri et al., 2001[42]).

### Gamma-aminobutyric acid (GABA)

Gamma-aminobutyric acid (GABA), an inhibitory neurotransmitter in the brain, its perturbation has been associated with MDD (Brambilla et al., 2003[9]). Investigating plasma GABA levels can provide insights into brain GABA expression, as peripheral GABA is derived from the brain (Petty et al., 1992[56]). Accumulating evidence suggests that both brain and plasma GABA levels are significantly lower in depressed patients compared to controls (Brambilla et al., 2003[9]; Sanacora et al., 1999[63]; Zheng et al., 2012[87]). Therapeutic interventions can also modulate GABA expression. For example, one study found that GABA levels decreased 2 hours after electroconvulsive therapy (Palmio et al., 2005[52]).

### Lysine

Lysine is the limiting essential amino acid in wheat and corn diets, lysine deficiency exists in developing countries where such diets are consumed frequently. The study of lysine in MDD is still emerging, a study comparing 25 patients with untreated depression before treatment with 25 controls found that lysine levels were reduced in MDD (Xu et al., 2012[82]). Although the mechanism is poorly understood, evidence suggests that lysine deficiency was related to a decrease in hippocampal norepinephrine release (Smriga et al., 2000[72]).

### Glutamine

Glutamine is a nonessential, highly abundant amino acid present in many tissues. Glutamine is involved in several processes including the cellular redox state (Labow and Souba, 2000[32]). Previous studies reported conflicting results regarding glutamine levels in patients diagnosed with MDD. Glutamine was found to be highly expressed in the cerebrospinal fluid glutamine levels in the depressed patients (Levine et al., 2000[34]). This finding was supported by another study (Mitani et al., 2006[47]) revealing that depressed patients had a significant elevation of plasma glutamine levels compared to control patients. In addition, higher plasma levels of glutamine were noted in depressed patients receiving antidepressant medication (Maes et al., 1998[39]; Mitani et al., 2006[47]).

This increase in glutamine could be as a compensatory mechanism against glutamate-induced excitability and neurotoxicity (Ramonet et al., 2004[60]; Sapolsky, 2000[65]). On the other hand, these results were challenged by a study (Zheng et al., 2012[87]), which found that plasma glutamine levels decreased in depressed patients compared to control subjects. However, other studies have reported no significant alteration in plasma glutamine (Altamura et al., 1995[1]; Mauri et al., 1998[43]).

### L-arginine

L-arginine is the precursor of nitric oxide (NOx). Although the role of NOx is extensively studied in MDD, however, less studies highlighted the potential role of L-arginine. Two previous studies demonstrated an increase in L-arginine in depressed patients compared to the controls (Mathis et al., 1988[41]; Mayoral-Mariles et al., 2012[44]), however these findings were not reproduced (Mitani et al., 2006[47]) who reported no changes in L-arginine levels. Other studied reported a decreased level of L-arginine in MDD (Hess et al., 2017[21]). 

### Glutamate

Glutamic acid is the main excitatory amino acid in the brain. Under pathological conditions it is known to be a potent neuronal cytotoxic through the activation of N-methyl-d-aspartate (NMDA) receptors (Ogawa et al., 2018[50]). Evidence showed the implication of glutamate in MDD based on the antidepressant effect of the anti-glutamatergic molecules such as ketamine (Krystal et al., 2002[31]; McGirr et al., 2015[45]; Woo et al., 2015[79]). Glutamatergic transmission abnormalities have been reported in plasma and cerebrospinal fluid of individuals diagnosed with depression. This increase in glutamate level could be attributed to the downregulation of glutamine synthetase, a glutamate degrading enzyme, found in the anterior cingulate of patients with MDD (Beasley et al., 2006[6]). Recently, the targeting of brain glutamatergic signaling has been the focus for treatment of MDD (Murrough et al., 2017[48]).

Several lines of evidence confirmed the elevation of glutamate in the plasma of individuals diagnosed with depression (Mauri et al., 1998[43]; Mayoral-Mariles et al., 2012[44]; Mitani et al., 2006[47]; Sanacora et al., 1999[63]; Tamborini et al., 2015[74]). Moreover one study identified high glutamate levels as a predictor for depression (Mayoral-Mariles et al., 2012[44]), this finding was further confirmed by a recent meta-analysis that confirmed a positive association between glutamate and depression (Huang et al., 2021[24]), moreover, glutamate plasma concentrated correlated positively with depression scales i.e. higher plasma glutamate was correlated with a higher depression score (Altamura et al., 1995[1]; Mitani et al., 2006[47]; Palmio et al., 2005[52]; Woo et al., 2015[79]). This increase may be explained by the release of glutamate from the brain to the blood due to disruptions in the blood-brain barrier. One study reported that ECT caused an increase in the plasma levels of glutamate (Palmio et al., 2005[52]), ECT may partially disrupt the blood-brain barrier (Chamberlin and Tsai, 1998[13]), in addition, plasma glutamate was increased in patients with stroke most probably due to the blood-brain barrier disruption (Castillo et al., 1996[12]). Interestingly, glutamate levels were normalized in respondents to SSRI (Maes et al., 1998[39]; Woo et al., 2015[79]); this finding is supported by previous studies suggesting the involvement of SSRIs in glutamatergic neurons (Schipke et al., 2011[68]; Wang et al., 2003[77]).

### Valine

Changes in serotonin levels have been associated with MDD, serotonin is synthesized from its precursor tryptophan. Administration of valine, a neutral amino acid prohibits tryptophan entry to the brain by competing for a transporter at the blood-brain barrier, therefore, lowering serotonin brain levels (Williamson et al., 1995[78]). In addition, one study found that valine lowered the prolactin response to D-fenfluramine, a serotonin-releasing agent, in healthy volunteers, which is an indirect evidence that valine may decrease brain 5-HT release in humans. Clinically, valine drink was not associated with depressive symptoms, however valine depressive effects were seen in remitted depressed patients (Delgado et al., 1990[17]; Williamson et al., 1995[78]). In accordance with these findings, one study reported that high platelet levels of valine at baseline could represent a negative prognostic factor of the depressive condition (Mauri et al., 2001[42]).

### Glycine

Glycine is an inhibitory amino acid neurotransmitter synthesized from serine (Iversen et al., 2009[27]; McNeil et al., 1994[45]). Glycine is an important metabolite in nitrogen metabolism which is implicated in MDD. Glycine plays an inhibitory role at the strychnine-sensitive glycine receptors (Malosio et al., 1991[40]); partial agonists of the strychnine-insensitive glycine receptors and competitive antagonist for NMDA receptors were associated with antidepressant-like action (Parsons et al., 1997[54]).

Few studies examined glycine plasma levels in patients diagnosed with MDD in comparison to controls, however, results were inconsistent. While some studies revealed that glycine levels were significantly higher in patients diagnosed with MDD versus controls (Mitani et al., 2006[47]; Palmio et al., 2005[52]), conversely, previous studies indicated either no significant difference or a decrease in plasma glycine levels (Altamura et al., 1995[1]; Maes et al., 1998[39]; Mauri et al., 2001[42]). Whether glycine can be modulated after treatment is yet to be uncovered. Glycine in one study was not changed hours after ECT (Palmio et al., 2005[52]), however, glycine levels were lower among responders to SSRIs (Ji et al., 2011[28]).

### Taurine

Taurine, a semi-essential amino acid is usually added to the infant milk formula. Taurine is one of the vital amino acids in the brain growth and development, it is essential in the neural plasticity, cell proliferation, differentiation and protection form excitotoxicity (Schaffer et al., 2014[66]). Taurine is thought to be an inhibitory neurotransmitter in the brain by activating GABAA receptors or strychnine-sensitive glycine receptors (del Olmo et al., 2000[16]; Ye et al., 1997[83]). Furthermore, taurine is involved in learning and memory (Li et al., 2017[35]; Rahmeier et al., 2016[59]). Taurine is transported to the brain through the blood-brain barrier and is distributed in the CNS thanks to the taurine transporter (Tamai et al., 1995[73]). The relationship between taurine and depression is controversial and not understood yet. Plasma taurine levels were found to be higher in depressed patients compared to controls (Altamura et al., 1995[1]). Moreover, antidepressant treatment significantly decreases plasma taurine levels (Maes et al., 1998[39]).

On the other hand, some evidence has demonstrated that taurine deficiency is related to depressive symptoms. In one animal model study, the supplementation of taurine leads to antidepressant-like effect in animals, this effect was associated with hormonal and neurotransmitter modulations (Wu et al., 2017[80]). Therefore, and based on this study it can be suggested to supplement adults with taurine to prevent depression given its safety profile.

### Methionine

Little evidence exists about the role of methionine in MDD, analyses from different set of patients have demonstrated a lower methionine plasma concentration in depressed patients compared to the controls (Ogawa et al., 2018[50]; Xu et al., 2012[82]). Methionine is catabolized to S-adenosyl L-methionine (SAMe), a molecule that received great attention due to its potential antidepressant properties. SAMe is registered in the United States as a dietary supplement and is marketed in many other countries for depression, fibromyalgia and other indications. Several studies investigated the antidepressant properties of SAMe versus placebo or antidepressants such as imipramine and escitalopram, however, according to a giant review by Galizia et al. (2016[20]), findings from these studies are yet inconclusive. Therefore, future high-quality studies and required.

### Alpha-aminobutyric acid (ABA)

Alpha-aminobutyric acid is a catabolic product derived from the metabolism of methionine, threonine, serine, and glycine (Ferrari et al., 2013[19]; Yudkoff et al., 1979[85]). The increase of ABA is a general non-specific marker that occurs with several medical conditions. The study of ABA levels in MDD is still emerging, one study has demonstrated that plasma ABA levels were shown to be high in depressed patients and were normalized after antidepressant treatment only in the responders' group. This can be explained by poor appetite, a depressive symptom, that is associated with a catabolic state that could lead to the enhanced catabolism of other amino acids thus leading to an increase in ABA levels (Woo et al., 2015[79]).

## Concluding Remarks

In conclusion, the role of plasma amino acids in major depressive disorder (MDD) has emerged as a significant area of investigation, shedding light on the complex relationship between biological processes and depressive symptomatology. The observed metabolic dysregulations, particularly in essential amino acids, glutamate-glutamine metabolism, and branched-chain amino acids, have potential implications as biomarkers for diagnosis and prognosis in MDD. Despite promising findings, utilizing plasma amino acids as therapeutic targets in MDD presents challenges. Inter-individual variability, genetic influences, gut-brain axis interactions, and gender differences necessitate further exploration to identify personalized treatment strategies that optimize therapeutic outcomes. Integrating plasma amino acid profiling with other biomarkers and clinical measures holds promise for improving diagnostic accuracy, treatment selection, and monitoring treatment response in MDD (Ogawa et al., 2018[50]).

To advance our understanding, unraveling the underlying molecular mechanisms linking plasma amino acids and MDD is imperative. Establishing causal relationships, elucidating specific pathways, and validating the clinical utility of amino acid profiling are crucial steps toward effective therapeutic strategies. Robust longitudinal studies, large-scale cohorts, and well-designed clinical trials are necessary to harness the full potential of plasma amino acids in personalized treatment interventions. Furthermore, integrating multi-marker panels and advanced statistical methods, such as machine learning algorithms, may enhance the precision and predictive value of amino acid-based biomarkers in MDD. These advancements will aid in developing tailored treatment approaches that consider individual patient characteristics and needs (Ogawa et al., 2018[50]).

By deepening our understanding of the intricate relationship between plasma amino acids and MDD, we can pave the way for innovative interventions targeting specific metabolic pathways. Incorporating amino acid-based therapies as adjunctive or personalized approaches may enhance treatment efficacy, improve remission rates, and alleviate the burden of MDD on individuals and society. In summary, investigating plasma amino acids in MDD offers promising avenues for advancing knowledge and improving clinical outcomes. However, further research is necessary to validate and expand upon these findings, ensuring their applicability and efficacy in the management of MDD (Qiu et al., 2023[58]).

## Notes

Omar Gammoh and Murtaza M. Tambuwala (Lincoln Medical School, University of Lincoln, Brayford Pool Campus, Lincoln LN6 7TS, United Kingdom; E-mail: mtambuwala@lincoln.ac.uk) contributed equally as corresponding author.

## Declaration

### Declaration of interest statement

The authors declare no conflict of interest.

### Data availability statement

The data that support the findings of this study are available from the corresponding authors, OG, MMT, upon reasonable request.

## Figures and Tables

**Table 1:**
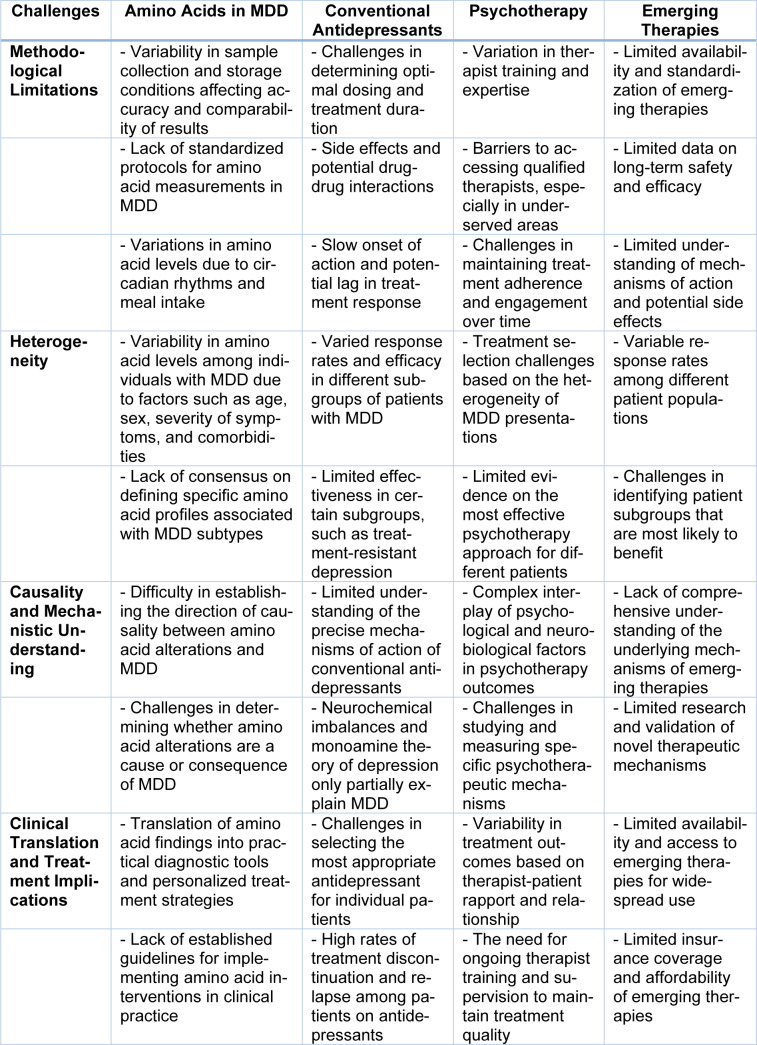
This table outlines the challenges associated with amino acid study in Major Depressive Disorder (MDD) and highlights the implications for conventional antidepressants, psychotherapy, and emerging therapies. It encompasses methodological limitations.

**Table 2:**
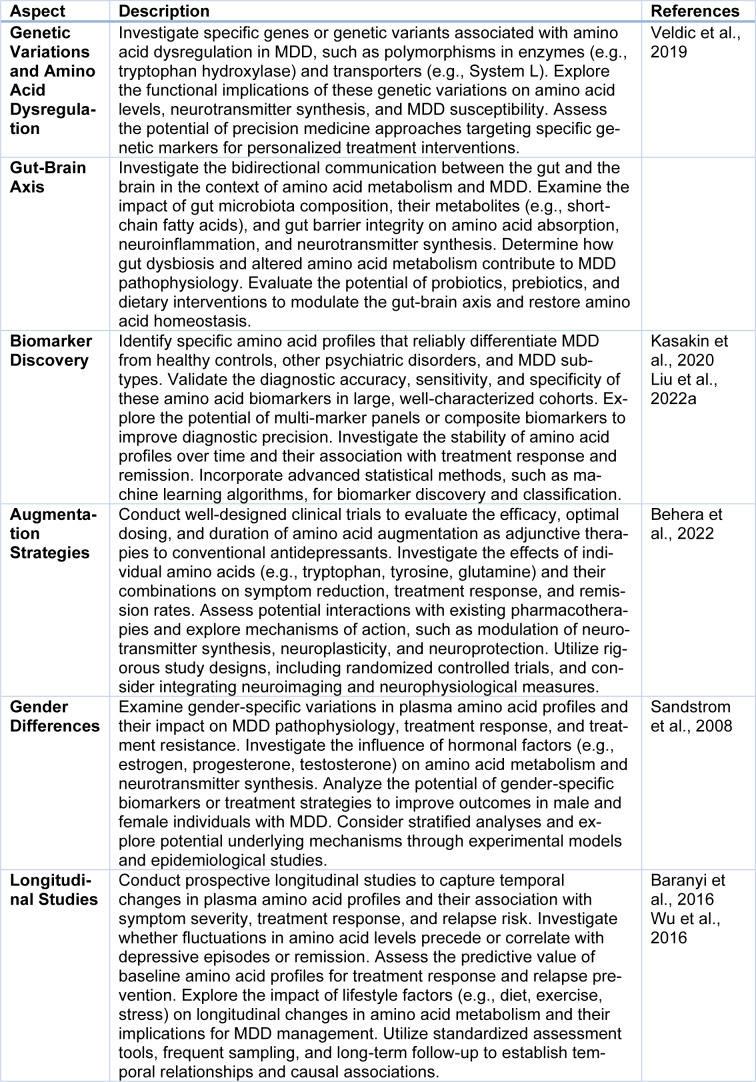
Future aspects
